# INTO THE WILD: EMBEDDING A COMPLEX, HOME-BASED DEMENTIA CARE PROGRAM INTO DIVERSE PRACTICE SETTINGS

**DOI:** 10.1093/geroni/igae098.1342

**Published:** 2024-12-31

**Authors:** Halima Amjad, Quincy Samus, Nancy Hodgson

**Affiliations:** Chair; Co-Chair; Discussant

## Abstract

MIND at Home® is a well-researched comprehensive care management program for community-living persons with dementia and cognitive impairment. Developed at Johns Hopkins University, the goal of the program is to provide holistic, family-centered care management by assessing and addressing a broad range of medical, social, and supportive care needs that place people with dementia and their care partners at risk for poor outcomes, institutionalization, and high care costs. Delivered by an interdisciplinary, dementia-proficient team, the program focuses on the home as the nexus of care and consists of specialized assessments, tools, and staff training. Memory Care Coordinators (MCCs) serve as the primary point of contact for program recipients and use a comprehensive needs assessment to develop individualized care plans. MCCs implement and monitor care plans and provide resource referrals, help with navigation and coordination of services and supports, teach problem-solving strategies, and provide dementia education, coaching, and emotional support to care partners. Managed care plans and advanced primary care are well positioned to implement this low-cost, high touch program. This session includes an introduction to the MIND at Home program and features three individual presentations by diverse implementation partners showcasing how the program was adapted and implemented in different health care environments (2 managed care plans-Texas and Pennsylvania; 2 large multispecialty clinics-Iowa and North Carolina). The session will wrap up with a summary of learnings from implementing the program “in the wild”, highlighting partner and stakeholder contributions to the evolution and successful implementation of the program in practice.

